# Imaging findings of IgG4-related kidney disease without extrarenal organ involvement: A case report

**DOI:** 10.1097/MD.0000000000016934

**Published:** 2019-08-23

**Authors:** ShuiXia Zhang, Qian Yang

**Affiliations:** Department of Radiology, Hubei Cancer Hospital, Tongji Medical College, Huazhong University of Science and Technology, China.

**Keywords:** diagnosis, IgG4-related kidney disease, imaging findings

## Abstract

**Rationale:**

IgG4-related disease (IgG4-RD) is a systemic chronic inflammatory disorder that can affect almost every organ. IgG4-RD includes IgG4-related kidney disease (IgG4-RKD), but lesions affecting the kidney alone or first are very rare, and a complete understanding is lacking. Computed tomography (CT) and magnetic resonance imaging (MRI) findings can show the typical characteristics of IgG4-RKD and provide information for accurate and rapid diagnosis.

**Patient concerns:**

We report a case of a 60-year-old woman who was admitted to our hospital for dizziness and instability while walking, her bilateral eyelids were also slightly swollen. She had no medical history.

**Diagnoses:**

CT and MRI images of the patient revealed multiple local and diffuse patchy lesions in the bilateral renal parenchyma and mass-like tissue in the bilateral renal pelvis, accompanied by right hydronephrosis. A pathological examination of renal samples showed numerous lymphocyte and plasma cell infiltration. Immunohistochemistry demonstrated approximately 50% of the IgG-positive plasma cells to be IgG4+. The serum IgG level was obviously elevated, with both C3and C4 levels were reduced. The patient was diagnosed with IgG4-RKD.

**Interventions:**

The patient received corticosteroid therapy at another hospital.

**Outcomes:**

The bilateral kidney lesions were smaller on follow-up CT images.

**Lessons:**

IgG4-RKD exhibits some characteristic imaging features. Despite the relatively low incidence of IgG4-RKD, it should be included in differential diagnoses when images show multiple lesions in kidneys with mild and delayed enhancement and hypointensity on T2WI in middle-aged to elderly patients

## 1 Introduction

IgG4-related disease (IgG4-RD) was first recognized in 2003 by Kamisawa et al.^[1]^ IgG4-RD can affect nearly every organ; the pancreas is the most common and typically the first identified. IgG4-related kidney disease (IgG4-RKD) was reported for the first time in 2004 as an extra pancreatic feature of autoimmune pancreatitis (AIP).^[2]^ However, lesions affecting the kidney alone or first are very rare. Here, we present a rare case of IgG4-RKD without affected extrarenal organs, providing a better understanding of its computed tomography (CT) and magnetic resonance imaging (MRI) characteristics.

## 2 Case report

**Figure 1:**
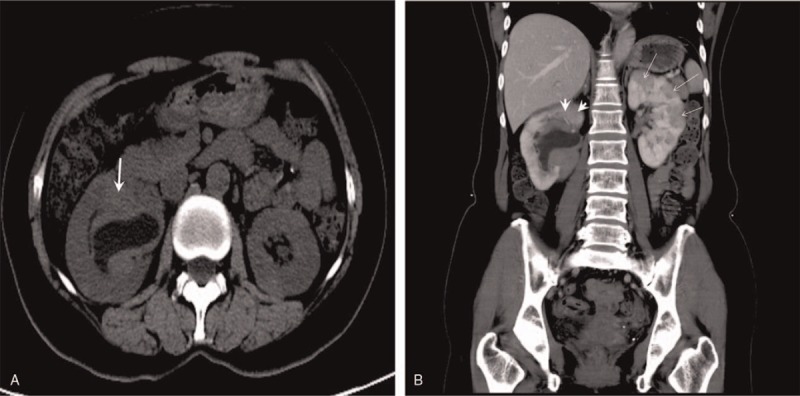
IgG4-RKD presentation on CT, displayed as a transaxial image precontrast (A) and coronal multiplanar reformation postcontrast (B). Precontrast image shows that the renal parenchyma lesions were not clear and that the right pelvic mass was slightly hyperdensity (thick arrow). Postcontrast image showed the left multiple renal parenchyma lesions were round, wedge-shaped, and with mild enhancement (arrows); the right renal parenchyma lesion showed diffuse patchy infiltration (arrowheads). CT = computed tomography, IgG4-RKD = IgG4-related kidney disease.

**Figure 2:**
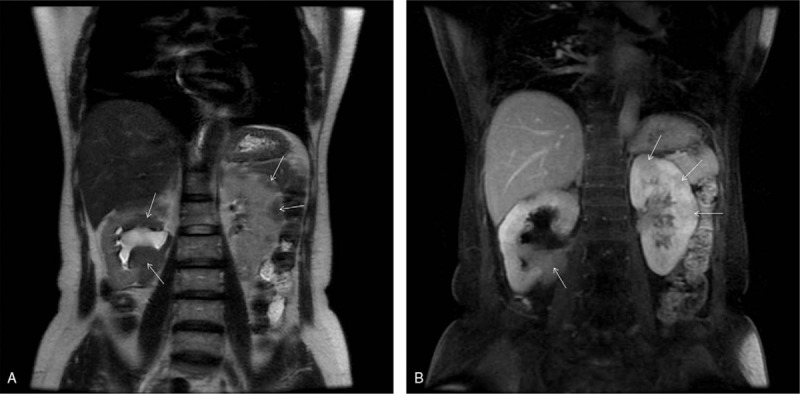
IgG4-RKD presentation on MRI, displayed as coronal T2WI (A) and postcontrast T1WI (B). The right pelvic mass and bilateral multiple renal parenchyma lesions were hypointense on T2WI compared with the normal renal parenchyma, with mild enhancement on contrast T1WI (arrows). IgG4-RKD = IgG4-related kidney disease, MRI = magnetic resonance imaging, T1WI = T1-weighted imaging, T2WI = T2-weighted imaging.

**Figure 3:**
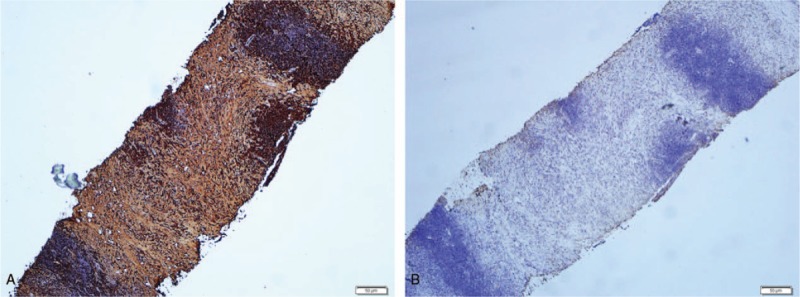
Histological images, presented as anti-IgG antibody staining (A), and anti-IgG4 antibody staining (B). Anti-IgG antibody staining showed numerous IgG+ plasma cells. Anti-IgG4 antibody stain showed an IgG4+/IgG+ ratio of approximately 50%.

A 60-year-old woman was admitted to our hospital for dizziness and instability while walking. A physical examination revealed that her bilateral eyelids were slightly swollen and that blood pressure was 148 mmHg/76 mmHg, with no other significant abnormalities. According to laboratory evaluations, her percentages of neutrophilic granulocytes and lymphocytes were 46.00% (normal value 50%–80%, slightly decreased) and 43.40% (normal value 20%–40%, slightly elevated), respectively. Indexes of liver function and urinalysis were within normal limits, and urinary cytology was negative. CT images (Fig. 1) and MRI images (Fig. 2) showed multiple local and diffuse patchy lesions in the bilateral renal parenchyma and mass-like tissue in the bilateral renal pelvis, accompanied by right hydronephrosis. Compared with the normal renal parenchyma, her lesions in the renal parenchyma and pelvis were isodense or slightly hyperdense on precontrast CT images (Fig. 1A) and hypointense on T2WI (Fig. 2A). The lesions also exhibited mild homogenous enhancement on both CT and MRI (Figs. 1B and 2B). There were no other abnormal findings for extrarenal organs. Because renal lymphoma was suspected, percutaneous renal biopsy was performed on the right renal pelvic mass. A pathological examination of the renal samples showed infiltration of numerous lymphocytes and plasma cells. Immunohistochemistry demonstrated that approximately 50% of the IgG positive plasma cells were IgG4+ (Fig. 3A and B). As IgG4-RKD was highly suspected, serological examination was recommended. The serum IgG level was 20.27 g/L (8–16 g/L) which was obviously elevated. Conversely, C3 and C4 levels were both reduced, at 0.56 G/L (0.9–1.5 G/L) and 0.08 G/L (0.2–0.4 G/L), respectively. IgA and IgM were within normal limits. The patient was diagnosed with IgG4-RKD and ultimately transferred to another hospital for receiving corticosteroid therapy. The bilateral kidney lesions were smaller on follow-up CT images.

## 3 Discussion

IgG4-RD is a systemic chronic inflammatory disorder. It generally affects middle-aged to elderly patients with a slightly male preponderance and can affect almost any organ, synchronously or metachronously.^[3–8]^ The pancreas, lacrimal and salivary glands, retroperitoneum, biliary tree, and kidneys are organs that are frequently involved. IgG4-RD has common histopathological features in affected tissue or organs, such as dense lymphoplasmacytic infiltration, marked IgG4+ plasma cell, and storiform fibrosis.^[9,10]^ Elevated serum IgG levels and hypocomplementemia are also characteristic features of IgG4-RKD, though up to 30% of patients with IgG4-RD have a normal range of serum IgG4 levels.^[10]^ In our case, the histopathological and serological findings all met the above conditions. IgG4-RKD may manifest as acute or chronic renal dysfunction because of IgG4-related tubulointerstitial nephritis (TIN) or sometimes hydronephrosis but seldom lead to hematuria or proteinuria, or cyturia. IgG4-RKD is usually accompanied by involvement of other organs,^[10,11]^ typically resulting in autoimmune pancreatitis (AIP), and approximately 35% of AIP patients suffer IgG4-RKD.^[12]^ Isolated cases of IgG4-RKD without other organ involvement are very rare. In one study, only 3 (6%) of 48 IgG4-RKD patients had renal lesions alone.^[13]^ IgG4-RD is a recently recognized disease, which that lacks full understanding and might be misdiagnosed in clinical work, particularly in patients with single-organ system involvement including IgG4-RKD. Some studies have suggested that IgG4-RKD shows different types of typical imaging characteristics.^[13–17]^ The lesions can be divided into 3 types based on their location^[13,16]^: the renal parenchyma, renal pelvic, and perinephric lesion. The renal parenchymal lesion is the most common subtype and is usually accompanied by TIN,^[15]^ followed by glomerular diseases. Each of the 3 types of lesions could appear alone or sometimes 2 types of lesions might appear together. The renal parenchymal lesions in IgG4-RKD can display 3 imaging patterns: round, wedge-shaped, and diffuse patchy infiltration lesions.^[13,16]^ In precontrast CT scan, the lesions are usually not clear and reveal mild enhancement in postcontrast and delayed reinforcement.^[3,13,16]^ The parenchymal lesions are often multifocal. On MRI T2WI images, the parenchymal lesions are hypointense compared with the normal renal parenchyma, and the dynamic enhancement pattern shown on post-T1WI is similar to that on postcontrast CT.^[13]^ Some studies report that the lesions are hyperintense on DWI, and this sequence has higher sensitivity for such lesions.^[18,19]^ Renal pelvic lesions manifest as diffused wall thickening of the renal pelvis or soft-tissue mass encasing the pelvis, and the lesions exhibit similar intensity (on T2WI images) and enhancement pattern compared with parenchymal lesions.^[13,14,17]^ Perinephric lesions are rare and appear as a rim-like lesion around the renal capsule, either diffuse or focal.^[13,20]^ All 3 types of lesions of the kidneys can be unilateral or bilateral. The shape of the involved kidney can enlarge in the early stage and become atrophied over time because of fibrosis. In our case, the right kidney showed slight atrophy, which means that the right kidney was at a late stage of the disease. In IgG4-RKD, hydronephrosis seldom occurs unless accompanied by retroperitoneal fibrosis entrapping ureter, even with pelvic lesions.^[13]^ In our case, the patient had mild right hydronephrosis without retroperitoneal fibrosis. This might be because the pelvic mass in the right kidney was huge.

IgG4-RKD has some characteristic imaging features. Despite the relatively low incidence of IgG4-RKD, it should be included in differential diagnoses when images reveal multiple lesions in kidneys with mild and delayed enhancement and hypointensity on T2WI in middle-aged to elderly patients, especially when coexisting with extrarenal abnormalities.

## Acknowledgments

Qian Yang, for providing the patient information; YiHao Yao, for correcting grammatical errors.

## Author contributions

**Resources:** Qian Yang.

**Writing - Original Draft:** Shuixia Zhang, Qian Yang.

**Writing - Review & Editing:** Shuixia Zhang.
